# A quantitative, hierarchical approach for detecting drift dives and tracking buoyancy changes in southern elephant seals

**DOI:** 10.1038/s41598-019-44970-1

**Published:** 2019-06-20

**Authors:** Fernando Arce, Sophie Bestley, Mark A. Hindell, Clive R. McMahon, Simon Wotherspoon

**Affiliations:** 1 Institute for Marine and Antarctic Studies, University of Tasmania, Private Bag 129, Hobart, 7001 Tasmania Australia; 2 Australian Antarctic Division, 203 Channel Highway, Kingston, 7050 Tasmania Australia; 3 Sydney Institute of Marine Science, 19 Chowder Bay Road, 2088 Mosman, New South Wales Australia; 4 Antarctic Climate and Ecosystems CRC, ACE CRC Private Bag 80, Hobart, 7001 Tasmania Australia

**Keywords:** Behavioural ecology, Marine biology

## Abstract

Foraging behaviour of marine predators inferred from the analysis of horizontal or vertical movements commonly lack quantitative information about foraging success. Several marine mammal species are known to perform dives where they passively drift in the water column, termed “drift” dives. The drift rate is determined by the animal’s buoyancy, which can be used to make inference regarding body condition. Long term dive records retrieved via satellite uplink are often summarized before transmission. This loss of resolution hampers identification of drift dives. Here, we develop a flexible, hierarchically structured approach to identify drift dives and estimate the drift rate from the summarized time-depth profiles that are increasingly available to the global research community. Based on high-resolution dive data from southern elephant seals, we classify dives as drift/non-drift and apply a summarization algorithm. We then (i) automatically generate dive groups based on inflection point ordering using a ‘Reverse’ Broken-Stick Algorithm, (ii) develop a set of threshold criteria to apply across groups, ensuring non-drift dives are most efficiently rejected, and (iii) finally implement a custom Kalman filter to retain the remaining dives that are within the seals estimated drifting time series. Validation with independent data sets shows our method retains approximately 3% of all dives, of which 88% are true drift dives. The drift rate estimates are unbiased, with the upper 95% quantile of the mean squared error between the daily averaged summarized profiles using our method (SDDR) and the observed daily averaged drift rate (ODDR) being only 0.0015. The trend of the drifting time-series match expectations for capital breeders, showing the lowest body condition commencing foraging trips and a progressive improvement as they remain at sea. Our method offers sufficient resolution to track small changes in body condition at a fine temporal scale. This approach overcomes a long-term challenge for large existing and ongoing data collections, with potential application across other drift diving species. Enabling robust identification of foraging success at sea offers a rare and valuable opportunity for monitoring marine ecosystem productivity in space and time by tracking the success of a top predator.

## Introduction

Foraging is a central element of an animal’s life. Being a successfully forager is directly translated into survival, reproduction, and ultimately population growth^1^. Foraging activity (where, when and how individuals acquire resources), is therefore a core concern that underpins ecological research. Acquiring this information from terrestrial systems is difficult but tractable. However, collecting information on foraging behaviour from marine animals is especially challenging because their oceanic environment limits our ability to make direct observations of feeding activities.

Broadscale approaches to studying the foraging ecology of marine predators include stomach contents^2^, stable isotopes^3^, fatty acid signature^4,5^ and genetic methods^6^. Animal telemetry approaches, with the on-going development and miniaturization of sensors, provides increasingly detailed insight into many aspects of marine organisms’ ecology^7^. Sensors currently devoted to directly studying foraging ecology of marine megafauna include stomach and oesophageal temperature sensors^8,9^, accelerometers capturing head or jaw movements^10–13^, as well as *in situ* miniaturised video cameras^14^. However, these approaches typically provide relatively observational short time-series on foraging behaviour in marine birds and mammals.

More commonly, telemetry-based studies of marine predators have relied on using behaviour to indirectly infer foraging. Generally, movement patterns of individuals are used to infer foraging areas. A broad suite of techniques have been applied to horizontal movements including heuristic methods such as area restricted search^15–17^ through to sophisticated process-based methods such as State-Space Models^18–20^. Research effort has also focussed on inferring foraging behaviour from vertical movements using dive-based indicators^21,22^. Nonetheless, direct empirical information on foraging events, and especially evaluating foraging *success*, remains elusive.

An alternative way to evaluate foraging success is to track changes in the animals body condition. For marine mammals, changes in body condition can be evaluated through buoyancy changes associated with an increase or decrease in the fat:lean tissue ratio^23^. Some marine mammals have been found to perform certain types of “drift” dives made up of three distinct phases: (i) an initial descent phase, when the animal is actively diving to depth, (ii) an inactive “drift” phase, when the animal is not actively swimming, and (iii) an ascent phase, when the animal actively returns to the surface. During the inactive phase, the rate of drifting is mostly determined by animal’s fat:lean tissue ratio and the surrounding media^23^. Buoyancy is known to be influenced by the density of the surrounding media and, in birds and mammals, by the effect of air in the lungs^24–26^. Biuw *et al*.^23^ investigated the effect of these parameters, finding only limited effects of salinity, and residual lung air at depths greater than 100 m. This type of dive was initially identified in Southern^27^ and Northern^28^ elephant seals, known to be deep divers^29,30^, but similar drift behaviours have been reported across a range of marine mammals including New Zealand Fur Seals^31^, sperm whales^32^, hooded seals^33^ and Baikal seals^34^. For the shallow diving species the effect of residual air in the lungs may influence the drift rate.

Southern elephant seals (*Mirounga leonina*) are an abundant predator of the Southern Ocean, spending over eight months per year at sea^27^. In between two periods on land, to breed and to moult, the seals travel long distances to forage^35^. As capital breeders, southern elephant seals fast during the periods they spend on land, so the energy they rely on for self-maintenance, moulting and breeding must be accumulated while the seals are at sea feeding; importantly it is these resources that are a key element determining individual fitness^36^. Southern elephant seals store energy in the form of lipids^37^ resulting in changes in the individual’s buoyancy as fat is accumulated or lost^23^. Quantifying these changes in buoyancy can provide an extremely useful index for determining foraging success, i.e., where and how much forage resource seals are acquiring, whilst at sea^23,38–42^.

Generally, dive profiles for determining the presence of drift dives come from high-resolution time-depth recording archival tags^42–45^. However, these tags have to be retrieved from the animal in order to access the high-resolution dive data. When these time-depth recorders are integrated into satellite-relayed tags, the data can be recovered in near real time without having to physically access the tag^46,47^. The most common way of recovering the dive information is through the ARGOS satellite system^48^. Despite the overall utility of the Argos system there are constraints on how much information can be received, so detailed time-depth profiles need to be summarized before being transmitted^46,47^. Mostly, time-depth profiles are summarized using a broken-stick algorithm (BSA)^49,50^. Although summarizing the data in this manner provides a reliable way of transmitting and receiving information, the reduced detail on the dive shape makes identifying dive types, including drift dives, challenging^51^.

Southern elephant seals are regularly tagged with satellite-linked time-depth-recorders across their range in the Southern Ocean. These tags normally carry oceanographic sensors to simultaneously record behaviour and physical hydrography^35,41,52,53^. As these tags are seldom recovered, most of the dive behaviour is only available from the summarized profiles transmitted through the Argos satellite system^54^. While drift dives can be detected from these^23,38,39^, changing dive profile summarization algorithms have prevented the widely use of methods based on summarized dive profiles to extract seal body condition. One recent study has proposed a new filtering process applied uniformly to all subsurface segments of summarized dives performed by the seals^41^. Here, we build on these approaches and develop a, flexible, hierarchically structured approach to identify drift dives and estimate the drift rate from summarized time-depth profiles that are increasingly available to the global research community (^54^ and references therein). In developing this new method we (i) automatically generate dive groups based on a ‘Reverse’ Broken-Stick Algorithm (RBSA), (ii) apply filters with threshold characteristics tuned for each group, ensuring non-drift dives are most efficiently rejected, and (iii) finally implement a custom Kalman filter to retain the remaining dives that are within the seal estimated drifting time series. Compared with available methods^41^, our approach is not solely based on a set of fixed criteria applied uniformly to all dive segments. Rather, we first generate a set of candidate drift dives, automatically grouped, allowing us to create a set of thresholds for each group. This makes our approach more flexible in terms of coping with diving heterogeneity. We apply these thresholds to specific groups of dives, rather to any diving segment. Our approach contributes to overcoming the long-term challenge for large existing and ongoing data collections which contain only summarized dive profiles, enabling robust identification of foraging success at sea with potential application across other drift-diving species. This will provide the basis for biological and environmental drivers of spatial and temporal patterns in feeding success to be further explored, a unique and rare opportunity in marine systems.

## Materials and Methods

### Tag data and processing

In developing our method to identify drift dives and estimate drift rates from summarized dive profiles, we first utilised high-resolution dive records. We randomly selected three high-resolution time-depth series from a set of Macquarie Island (50°30′S, 158°57′E) deployments on southern elephant seals during 2004. All tags were velocity-time-depth recorders (VTDR, Wildlife Computers MK8, Redmond, Washington, USA) sampling depth and velocity at 30 s intervals. Tags were attached to adult females; two during post breeding and one during the post-moult trip (see^55–58^ for a full description of the general field procedures). Seal instrumentation was carried under ethics approval form the Australian Antarctic Animal Ethics Committee (AAS 2265 & AAS 2794) of the Australian Antarctic Division.

**Figure 1:**
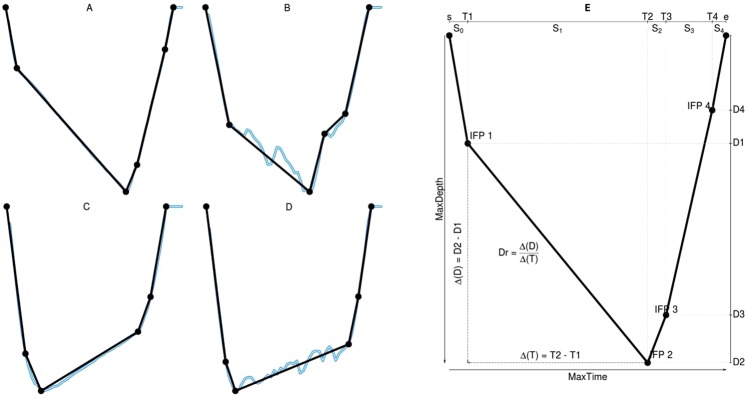
Explanation of drift dives. Obtained from summarized high-resolution tag data. Example of an (**A**) negative and (**C**) positive drift dive, as well as non-drift dives whose summarized forms incorrectly resemble (**B**) negative and (**D**) positive drift dives. Blue lines represent high-resolution time depth profiles, while black represents the summarized profiles from the Broken-Stick algorithm. (**E**) Diagram of a summarized drift dive including the main criteria used to classify summarized profiles as drift dives. For this dive, the ifp (inflection point order) is 2.1.3.4. Summarized inflection points are IFP1{T1, D1}, IFP2 {T2, D2}, IFP3 {T3, D3} and IFP4 {T4, D4}. ps0 represents the proportion of the dive duration spent on the descending phase (T1/MaxTime etc.). S_1_ the proportion spent along the first BSA segment (T2 − T1/E), S_2_ along the second segment (T3 − T2/E), S3 for the third segment (T4 − T3/E) and S4 between the last ifp and the end of the dive (e − T4/E). Drift rate (Dr) is calculated as the difference in depth divided by the difference in time over the drifting segment (in this case, the segment between IFP1 and IFP2).

From all the dives (n = 18064) recorded by the tags, we only kept those dives reaching a minimum depth of 100 m, to avoid potential bias from residual air in the lungs. We also removed those dives with a duration lower than 300 sec as they are short, shallow, exploratory dives^39,44,59^ that resulted in a final set comprising 97.5% of the original dives (n = 17622, Table 1). We visually inspected all dives meeting these criteria and classified them as potentially drift or non-drift dives according to their shape and the velocity records; potential drift dives requiring a passive ascent or descent phase, without directional changes (“wiggles”) (Fig. 1) or abrupt changes in the recorded velocity. Drift dives were further allocated as certain or uncertain, and as positive or negative. We then summarized each high-resolution VTDR dive using a Broken-stick algorithm (BSA). This reproduces the on-board processing of dives that occurs on the SRDLs^49,50^, resulting in a summarized form with only four subsurface inflection points retained together with the start and end points (Fig. 1).

**Table 1:** Number of high-resolution time-depth recorder (TDR) dives used for the development of the drift dive methodologies.

Seal id	Trip	Dives	Drift dives			
			Certain	Uncertain	Positive	Negative
b14304pm	pm	10913	703 (6.4%)	180 (1.6%)	178 (1.6%)	525 (4.8%)
c06404pb	pb	3879	179 (4.6%)	19 (0.5%)	0 (0%)	179 (4.6%)
c09004pb	pb	2830	190 (6.7%)	51 (1.8%)	0 (0%)	190 (6.7%)
Total		17622	1072 (6.1%)	250 (1.4%)	178 (1.0%)	894 (5.1%)

Shown are the numbers of dives visually classified as drift dives; either as certain or uncertain. Certain drift dives are indicated as positive (i.e. upward drift segment) or negative (i.e. downward drift segment). Trip types are indicated as post-moulting (pm) or post-breeding (pb). Numbers in parentheses give percentages.

### Drift dive selection process

For the automated drift dive selection process on the summarized dive profiles, we introduce two new important steps. First, we pay particular attention to the order in which the inflection points are selected by developing a ‘Reverse’ Broken-stick algorithm (hereafter RBSA), similar to a recently implemented approach^49^ which aids in grouping candidate drift dives. Secondly, we develop a set of threshold criteria with respect to dive profile characteristics to apply across groups, to automatically select those dives whose drift rates will be submitted to the final Kalman filtering stage.

#### Reverse Broken-Stick algorithm (RBSA)

The RBSA generates the order in which the inflection points are selected before the satellite transmission. The basis for the RBSA is the same as the original BSA. From the summarized profiles the only inflection point whose original position is known is the deepest point, that is, the first selected point (as it shows the highest difference between the original high-resolution time-depth profile and the surface). A linear profile is constructed between this deepest point and the start and end points of the dive. The second point will then be determined by the largest discrepancy between the reconstructed profile and the transmitted points. The RBSA recursively reconstructs the profile until the fourth and final point is found^49^. The RBSA also generates the original residuals from the BSA the last of which, *i.e*. the largest remaining difference between the summarized and detailed profile, gives a relative indication of the amount of vertical activity not well captured by the summarized profile.

The inflection points are transmitted sorted by time of occurrence along the dive, not by the order in which they are selected by the BSA. Thus, an inflection point order of [2.1.3.4] indicates that the first point selected by the BSA will be the second timestamp (T2, D2) of the dive profile, the second inflection point selected is the first timestamp (T1, D1) and so on. This inflection point order (ifp) is used to organise the dives into groups.

**Figure 2:**
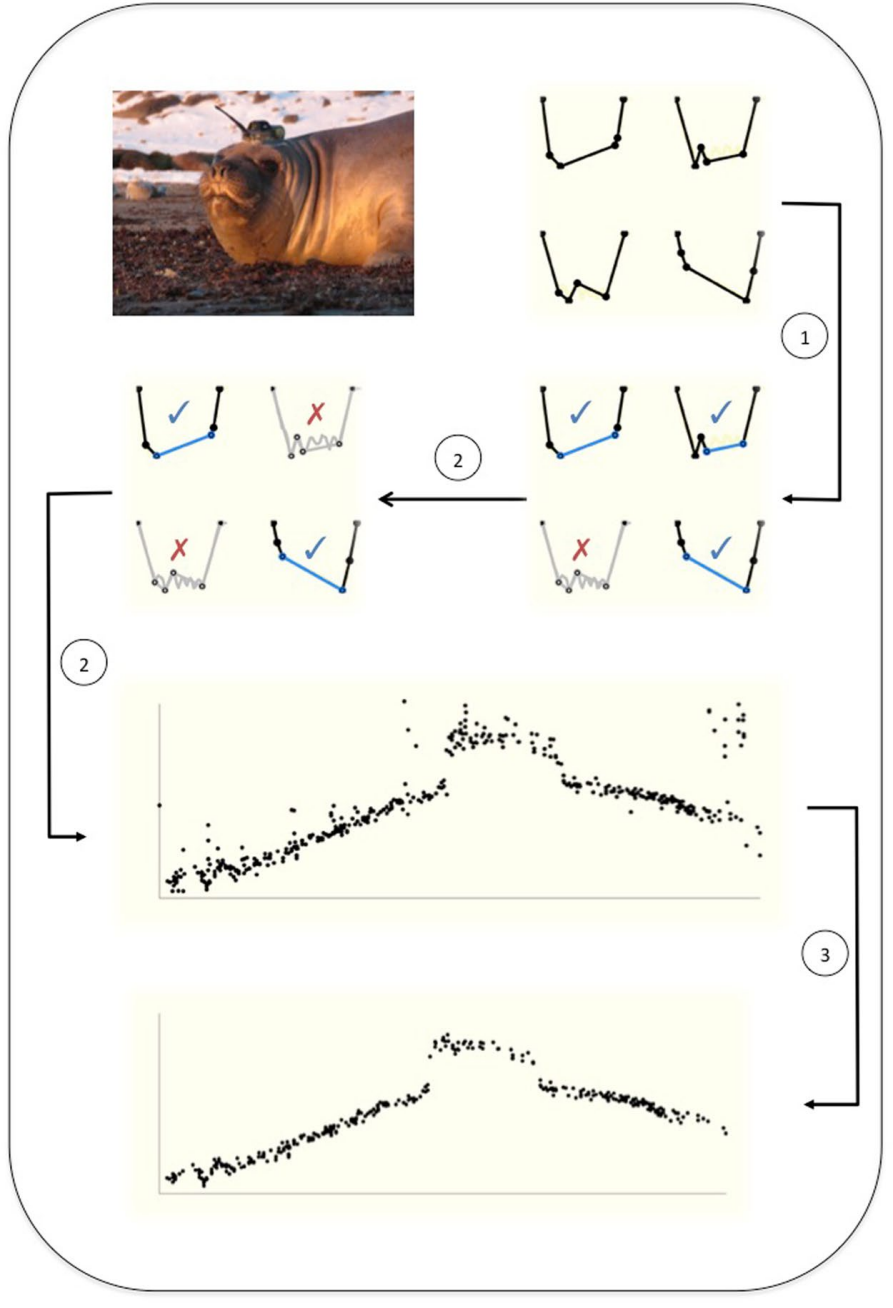
Diagram representing the drift dive methodology: i. Seal is instrumented and summarized dive profiles are transmitted; ii.1. Using the Reverse Broken-Stick Algorithm (RBSA), dives are grouped according to inflection point ordering and candidate groups of drift dives identified. At the same time, the putative drift segment is assigned (blue); ii.2. A set of threshold criteria are applied to each candidate group to further isolate certain drift dives, however visualization of the observed drift rates reveals some noise remains in the drift trajectory; iii: The custom Kalman filter is applied to the drift rate observations to obtain the final drift rate trajectory over time. Seal picture© Fernando Arce.

The distribution of known (certain) drift dives from the high-resolution VTDR data was checked across these RBSA groupings, and used to make a first pre-selection of candidate drift dives (i.e. those dives that may be drift dives). Of the possible 24 groups identified by the inflection point ordering, eight comprised the majority of the known drift dives (>90% total). These groups became the only ones considered as potential drift dives (step 1, Fig. 2) and retained for the following calculations and selection procedures.

#### Drift rate estimate

A combination of different dive proportions and the position of the deepest point are then used to determine the drift segment of each potential drift dive (Appendix A and Table 2). Drift rate (m s^−1^) is then calculated as the difference of depth between the start and end point of the drift segment with respect to the time spent on the segment, i.e. Dr = ∆(D)/∆(T) (see Fig. 1). The sign of the drift rate allows us to further allocate dives into negative/positive subgroups.

**Table 2:** Eight main RBSA groups identified by the inflection point ordering which comprised the majority (90.5%) of drift dives.

Order	Drifting segment		
	1	2	3
2.1.3.4	mdepthbias > 0	mdepthbias < 0	
2.1.4.3	ps1 > 25	ps1 ≤ 25 & (1.1 × ps2) ≥ ps3	ps1 ≤ 25 & (1.1 × ps2) < ps3
	mdepthbias < 0 & ps1 > ps3		
2.4.1.3	*or*	mdepthbias > 0 & ps1 ≤ ps2	mdepthbias < 0 & ps1 ≤ ps2
	mdepthbias > 0 & ps1 > ps2		
3.1.2.4	avratio < 0	avratio > 0	
		ps1 < 25 & s <0 & t > 0	ps1 < 25 & s > 0 & t < 0
3.1.4.2	ps1 > 25	*or*	*or*
		ps1 < 25 & s < 0 & s < 0 & hp2 > hp3	ps1 < 25 & s < 0 & s < 0 & hp2 < hp3
3.2.1.4		All	
3.4.1.2	mdepthbias > 0 & ps1 > ps2	mdepthbias > 0 & ps1 < ps2	mdepthbias < 0
4.2.1.3	mdepthbias > = 0		mdepthbias < 0

The criteria shown are those used to determine the drifting segment of the candidate drift dives within groups. All dives of the 3.2.1.4 group have the same drifting segment (segment 2) so no criteria is required to determine it. {f, s, t} are the change of depth with respect to time for the first, second, and third segments (excludes the initial/descendant, and last/ascent segments).

#### Developing threshold criteria

For each individual dive profile, a number of numerical variables were calculated based on dive proportions. Proportional values were used to scale variables irrespective of shorter/longer or shallower/deeper absolute profiles, to minimize the influence of seals diving variability. Figure 1 provides a visual aid for these variables, which each give information about the dive shape, and a detailed description of the threshold criteria is provided in Appendix A. For each dive group we separately constructed density plots of these variables for both certain drift and non-drift dives, allowing different threshold criteria to be developed and automatically applied across the different groups (step 2 in Fig. 2).

The criteria selection and its thresholds for each group were developed sequentially as follows:

First, density plots of all the proposed criteria for each seal were constructed, to show the degree of overlapping for drift and non drift dives. The criterion with the lowest degree of overlap was then selected first. This selected criterion was inspected in greater detail with an accepting-rejecting plot (see Appendix A) to find an optimal threshold; aiming for a reduction of ~50% of the non drift dives at a cost of losing as much as 5–10% of the true drift dives. Once the first threshold was identified, it was applied to the dataset, and the previous step was iterated for each of the 15 groups until no further optimal threshold could be found.

### Kalman filtering drift rates

The two-step process described above supplies a final set of candidate drift rates to a custom Kalman filter. This is implemented to remove those dives with unrealistic drift rates in relation with the seal drift time series. Step 3 in Fig. 2 gives a schematic representation of the filtering process. Kalman filters are a family of methods used to filter time series and reject/recalculate points using the trajectory of the signal, for example to filter noisy animal movement paths^60^. We applied the Kalman filter to the drift rate time series of each individual animal. Our Kalman filter assumes that (i) the vertical drift rate of a seal is proportional to the squared root of the difference between water density and the seal body density, (ii) water density is constant, and (iii) seal density changes through mass accretion (primarily blubber), and takes the general form:

$${\rho }_{k}=\frac{{m}_{0}+{\delta }_{k}}{{v}_{0}+V{\delta }_{k}}$$

$${\mu }_{k}=\alpha \,sign\,({\rho }_{k}-1)\sqrt{|{\rho }_{k}-1|}$$

where:

*ρ*_*k*_ = seal density at dive k

*m*_0_ = initial mass

*δ*_*k*_ = mass increment

*v*_0_ = initial volume

*Vδ*_*k*_ = increment of volume associated with the mass increment at dive k

*μ*_*k*_ = buoyancy of the seal at dive k

*α* = *constant*

*sign*(*ρ*_*k*_ − 1) = sign of the seal’s density (it is lost on the square root calculation)

$\sqrt{|{\rho }_{k}-1|}$ = squared root of the difference between seal density and water density at dive k

We then modelled the mass increments *δ*_*k*_ as a random walk:

$${\delta }_{k}={\delta }_{k-1}+{\eta }_{k}$$

$${\eta }_{k} \sim N(0,{\tau }^{(\delta )}/({t}_{k}-\,{t}_{k-1}))$$

where:

*δ*_*k*_ = mass increment at dive k

*δ*_*k*−1_ = mass increment atdive k − 1

*η*_*k*_ = variation on the mass increment associated with the dive k

*τ*^(*δ*)^ = variance of η_k_ dependent on the masss

(*t*_*k*_ − *t*_*k*−1_) = timeincrement between the current dive and the previous one

and we consider the error on the drift rate observations as normally distributed:

$${r}_{k} \sim N({\mu }_{k},{\tau }_{{z}_{k}}^{(r)})$$

where:

*r*_*k*_ = observed drift rate for the dive k

*μ*_*k*_ = buoyancy of the seal at dive k

${\tau }_{{z}_{k}}^{(r)}$ = variance on the observed drift rate conditioned on whether or not dive k belongs to the trajectory

The Kalman filter evaluates whether any drift rate observation associated with the time-varying density change process is inside or outside the most likely trajectory of the time series based on the expected variation associated with both the process and the observation. Whether any potential drift dive is inside or outside the trajectory of the drifting time series is defined as a binomial state variable Z_k_ with two possible outcomes: 1 (dive k is inside the trajectory) or 0 (dive k is outside the trajectory):

$${z}_{k} \sim Bin(1,p)$$

The most probable drift rates of observations that are unlikely to be inside the trajectory of the drifting time series may also be estimated. However rather than using the estimated drift values, we accept as drift dives only those with a probability of being inside the trajectory close enough to 1 [P(Z_k_ = 1) > 0.95] and retain these observed drift rates.

### Validation of the method

#### Drift rate evaluation

To obtain the “true” drift rates we computed the rate of change in depth for all time-steps inside each drifting segment from the original high-resolution time-depth records. To check the robustness of our final drift rates the median of all these values was then subtracted from the value extracted from the summarized dive profile as a measurement of bias.

We also generated an observed daily averaged drift rate (ODDR) from the high-resolution drift dives. These were compared with a daily averaged drift rate calculated from the summarized profiles using our method (SDDR), and the difference between both used as another direct measure of bias.

To quantify the improvement in estimations due to the Kalman filter we calculated the SDDR before and after the application of the Kalman filter. We compared the performance by calculating the squared error and its mean (msr) between both SDDR’s and the ODDR.

#### Validation with independent seal data

To assess our model performance we processed six additional data sets from Macquarie Island deployments during 2004 post-breeding trips as well as four from 2005 post-moulting trips (n = 10 seals). We visually inspected the high-resolution profiles of those dives accepted by our hierarchical procedures, and calculated the proportion that were true drift dives.

## Results

From the 17622 high-resolution dive profiles visually classified, 1072 (6.1%) and 250 (1.4%) were classified as certain or uncertain drift dives, respectively (Table 1). Seal b14303, undertaking a post-moult trip, was the only one with identifiable certain positive drift dives, with a proportion of 4.8% and 1.6% for negative and positive drift dives respectively (Table 1). We found an average of 5.1% (range 4.6–6.7%) of certain negative drift dives across the three seals (Table 1). These numbers are consistent with previously reported values from this dataset^39^.

### Drift dive selection process

Upon application of the RBSA, we retained eight major dive groups as candidate drift dives with the following inflection point (ifp) orders: [2.1.3.4, 2.1.4.3, 2.4.1.3, 3.1.2.4, 3.1.4.2, 3.2.1.4, 3.4.1.2 and 4.2.1.3]. This removed 3909 out of the 17622 dives, of which only 121 were considered certain drift dives i.e., an acceptably low (3.1%) overall false rejection rate (Appendix B). The eight retained groups together represented more than 90% of the certain negative drift dives and 86% of the certain positive drift dives (Appendix B). The criteria developed to automatically determine the drifting segment for each of the eight retained dive groups are shown in Table 2. As an example, for group [2.1.4.3] if the proportional dive time occupied by segment 1 is above 0.25, this comprises the drift segment; otherwise the drift segment comprises the next longest segment.

For each group, up to seven threshold criteria were applied sequentially to give a criteria-threshold combination that efficiently rejected certain non-drift dives. The specific criteria applied to each dive group and their threshold values are reported in Table 3. Positive drift dives occurred throughout all major groups, excluding [3.2.1.4] which represented only negative drift dives, and different criteria were applied between positive and negative drift dives within groups (Table 3). An example of a widely applied criterion is *d1*, the ratio between the depth of the first inflection point and the maximum depth, which for known drift dives was less than 0.6 to 0.8 across all groups. The exception was group [3.2.1.4] in which the drift segment is always segment 2 so this threshold (0.8) applies instead to the *d2* criteria. After the application of the threshold criteria, 615 out of 17622 (i.e. 3.48%) candidate drift dives were retained for the Kalman filtering step.

**Table 3:** Threshold values for dive-based criterion applied to the eight main RBSA groups.

RBSA order	2.1.3.4		2.1.4.3		2.4.1.3		3.1.2.4		3.1.4.2		3.2.1.4	3.4.1.2		4.2.1.3	
Dive sign	**−**	**+**	**−**	**+**	**−**	**+**	**−**	**+**	**−**	**+**	**−**	**−**	**+**	**−**	**+**
t1		(0.7, 0.14)	<0.14	<0.15	<0.14	<0.14	<0.14	<0.14		<0.12		<0.14	<0.12	<0.9	<0.8
d1	<0.8		<0.7		<0.6	<0.8	<0.6	<0.85	<0.8			<0.8		<0.8	
d4			<0.8	<0.6		<0.8		<0.7	<0.8			<0.8	<0.8	<0.8	
mrratio			<0.15	<0.2		<0.15				<0.3	<0.2	<0.2		<0.2	
ps1	>0.4		>0.4		>0.4		>0.4	<0.15	>0.4						
t4		>0.9								>0.9			>0.85	>0.85	>0.8
mdepthr						(0.6,1.4)		(0.8,1.5)		(0.8,1.3)					(0.8, 2)
sratio	<10								<10				(2,7)		
ps2								>0.4		>0.2	>0.45				
ps3												<0.2			
sdd		(0.13, 0.4)								(0.1, 0.3)					
r1						<0							<0		
r4		<0													
mdepthbias				<0											
meand						<0.8									
d2											<0.8				

Only the cells of the criteria applied contain values. Values in brackets represent the lower (left) and upper (right) open thresholds of the threshold acceptance interval. Dive sign indicates criteria applied to negative (−) or positive (+) drift dives within groups. For full criteria description see Appendix A. In brief: {d1, d2, d3, d4} = ratio between the depth of the first, second, third and fourth inflection points and the maximum depth. {ps1, ps2, ps3} = proportion of the dive duration spent on the first, second, and third segments generated by the RBSA. sratio = ratio between the vertical rate of the descending phase and the vertical rate of the first segment post-descent. meand = mean value of {d1, d2, d3, d4} described above. sdd = standard deviation of {d1, d2, d3, d4}. {r1, r2, r3, r4} = residuals obtained by fitting a least square linear regression through the four inflection points {D1, D2, D3, D4}. mrratio = ratio between the smallest BSA residual and the maximum depth. mdepthbias = difference between the time at maximum depth and half of the total dive duration. mdepthr: ratio between the averaged depth of the inflection points {D1, D2, D3, D4} and the maximum depth. {t1, t2, t3, t4} = ratio between the time of each inflection point and the dive duration.

### Validation

**Figure 3:**
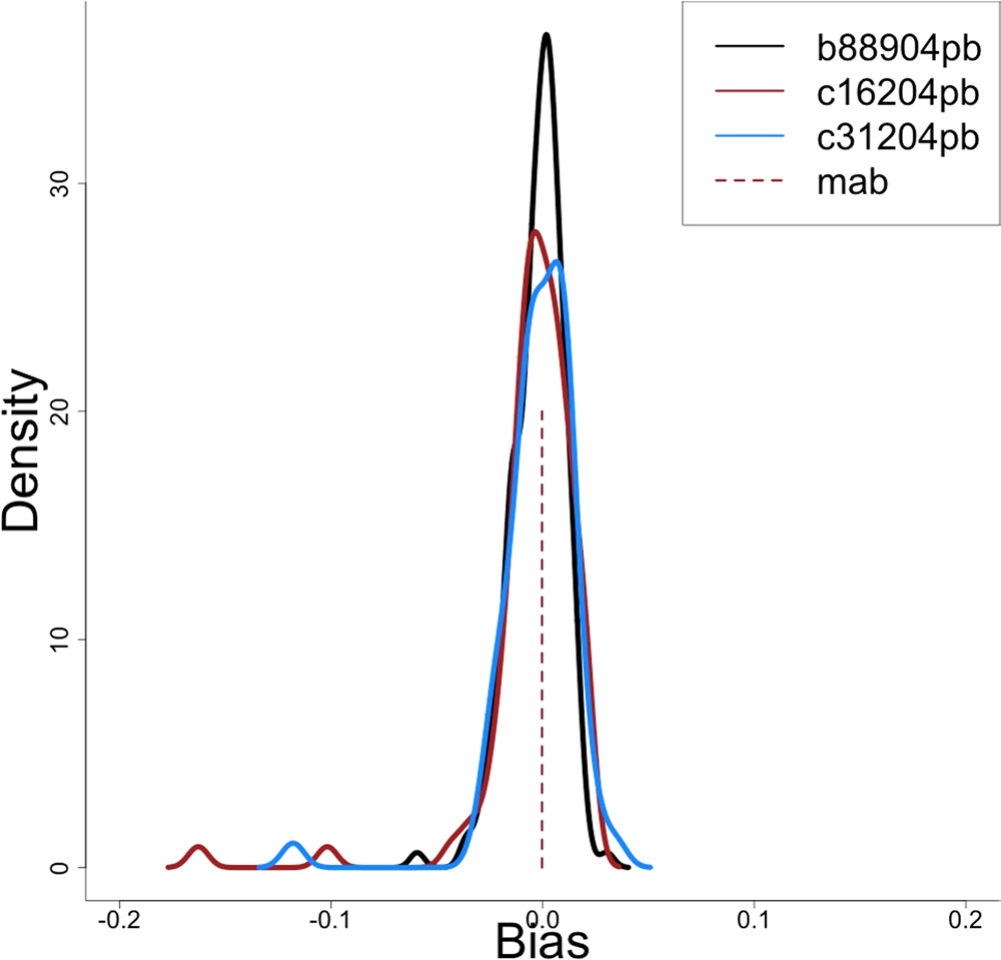
Drift rate evaluation. Density plot shows the bias calculation for the final drift rates obtained using summarized profiles relative to the “true” drift rates obtained from high-resolution data. Curves are shown for the three processed seals (n = 735, 191 and 200 drift dive observations), together with the median averaged bias (mab = −0.0003).

Application of the Kalman filter rejected 155 (28.2%), 57 (34.1%) and 66 (37.3%) of the final candidate drift dives for the three test seals. The comparison of our final post-filter drift rates with the “true” rates calculated from high-resolution profiles showed there were no differences in the bias distribution among the three seals (F_238_ = 0.3893, p = 0.6778, Fig. 3). The median bias after pooling the three bias distributions across the three seals was -0.0025 (S.D: 0.07, 95% confidence interval (CI): −0.03, 0.02).

**Figure 4:**
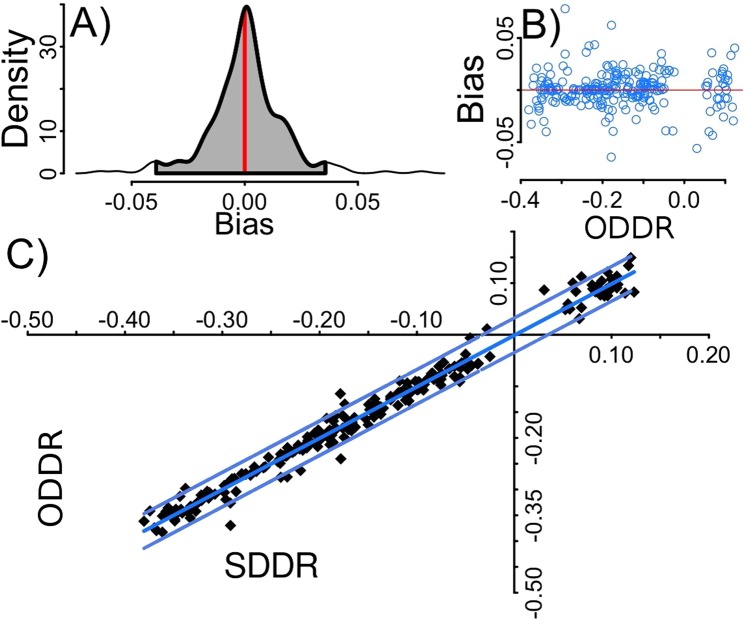
Validation of the method. (**A**) The density distribution of the calculated bias between the daily averaged drift rate from summarized data (SDDR) and the observed daily averaged drift rate (ODDR) for the three seals. Grey shadowed area covers the 95% confidence interval, and vertical red line is drawn at the median. (**B**) The calculated bias versus the ODDR, evidencing a lack of any trend (horizontal red line set at Y = 0). (**C**) The positive linear relationship between the ODDR and the SDDR and the 95% confidence interval (SDDR = −0.001 + 0.986ODDR, r^2^ = 0.984).

Based on the comparison between the observed daily averaged drift rate (ODDR) and that obtained using our method for summarized profiles (SDDR) there were also no differences in the bias values across the three seals (F_238_ = 2.61, p = 0.08). Once pooled the median value for the bias did not depart significantly from 0 (median: 0, S.D: 0.02, 95% CI: -0.001, 0.002; t_238_ = 0.58, p = 0.56; Fig. 4A). There was no evidence for any trend in bias magnitude associated with an increase in the theoretical daily averaged drift rate (r = 0.02, t_238_ = 0.33, p = 0.74; Fig. 4B). The correlation between the ODDR and SDDR daily averaged drift rates (r = 0.99, p < 0.001; Fig. 4C) indicates our method was highly successful for the test seals.

**Figure 5:**
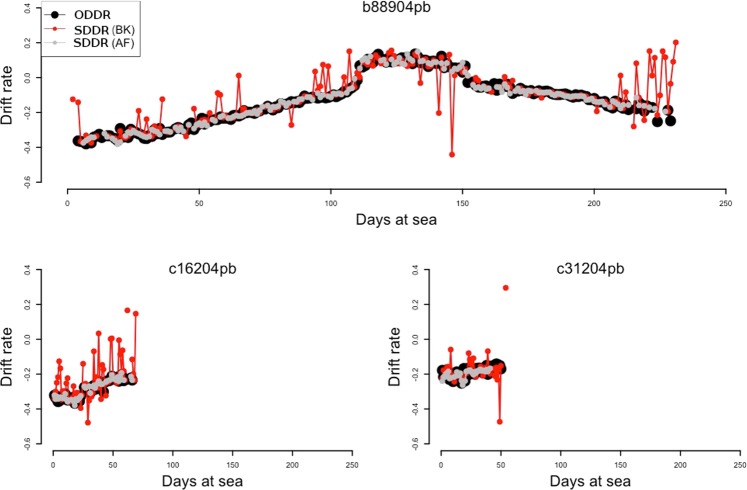
Kalman filter application. Comparison shows three daily averaged drift rate trajectories of the seals used to develop this method (b88904pb, c16204pb and c31204pb). ODDR refers to the observed daily averaged drift rate and SDDR to the daily averaged drift rate from summarized data both before (BK) and after (AK) applying the Kalman Filter. Lines between points join consecutive daily estimates.

**Figure 6:**
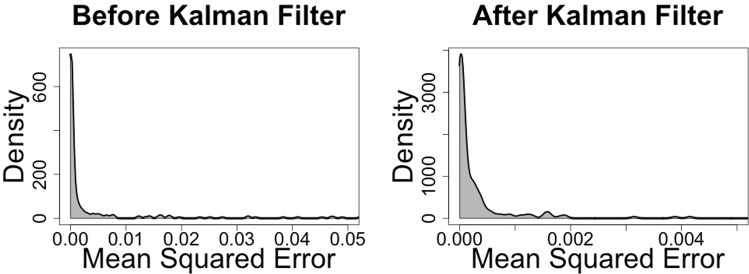
Kalman filter performance. Mean squared error (msr) between the summarized daily drift rate (SDDR) and the observed daily drift rate (ODDR) across all observations (n = 1126). (**A**) Before the use of the Kalman Filter (mean ± SD = 0.005 ± 0.014, upper 95% CI = 0.04), and (**B**) after the Kalman Filter’s application (mean ± SD = 0.0002 ± 0.0006, upper 95% CI = 0.0015). Note the order of magnitude reduction on the x-axis scale in (**B**).

After applying the Kalman filter, the SDDR time series efficiently followed the ODDR on all three test seals (Fig. 5). The Kalman filter implementation substantially reduced the mean squared error between the SDDR and the ODDR by an order of magnitude (95% upper CI before and after being 0.04 and 0.0015, Fig. 6).

The validation of our approach with 10 independent seals showed on average the percentage of retained dives being true drift dives was 87.5% (S.D: 9.35, Table 4).

**Table 4:** Validation of the drift dive methodology with 10 independent Macquarie Island seals.

Seal id	Trip	N	Rd	%d	RDd	% Dd
b88904pb	pb	4376	72	1.65	66	93.05
c16204pb	pb	6287	87	1.38	86	98.85
c31204pb	pb	5848	68	1.16	61	89.7
c69904pb	pb	2867	197	6.87	180	91.37
c79004pb	pb	4828	80	1.66	68	85
h28504pb	pb	3921	64	1.63	60	93.75
c16305pm	pm	11159	240	2.23	160	66.66
f99305pm	pm	10034	220	2.27	171	77.72
h23305pm	pm	12331	732	6.09	717	97.95
h83305pm	pm	10011	268	2.74	225	83.95
Total		77179	2246	2.82	1972	87.8

Seal id = reference code for each individual tag/seal. Trip: pb = post-breeding trip, pm = post-moulting trip. N = Total number of dives recorded by each tag. Rd = number of retained dives after the application of our method. %d = proportion of dives retained from the total number of dives recorded. Rdd = number of retained drift dives. %Dd = proportion of the retained dives that were true drift dives, as determined by visual inspection of all retained dives using the original high resolution time-depth profiles.

The full filtering process as applied across the test (n = 3) and validation (n = 10) seals is visualised in Appendix C. All the filtering procedures have been implemented in R^61^ and JAGS^62^ and are freely available in the form of an R package (https://github.com/farcego/SlimmingDive).

## Discussion

The occurrence of drift dives, where animals passively sink or rise in the water column, enables buoyancy changes to be determined in some marine species. Drift rate changes related to fat gain (or loss) provide a rare and valuable index of foraging success at sea. Here, we have presented a, reliable method to quantify drift rates from the summarized satellite relayed time-depth-record data widely used for migratory marine species. The process-based Kalman Filter is consistent with our understanding of the ecological processes governing the energy budgets of elephant seals i.e. a gain in fat during the two at-sea phases of the seals annual cycle, with effective results for both post-breeding and post-moult animals. We have not directly considered the effects of residual air as a potential source of bias on our estimates because (i) we don’t consider shallow dives (i.e. less than 100 m depth) as potential drift dives, and (ii) elephant seals exhale before diving. Previous research^23^ evaluated the potential effect of residual air present in elephant seal lungs, finding little effect on deep dives. That may not be the case for other shallower, breath-holding marine mammals, where these assumptions may be too strong. Our method overcomes a long-term challenge to robustly identify at-sea foraging success, and provides great opportunity for linkages between ecology, physiology, behaviour and environmental drivers to be further explored.

The new method provides a time series of drift rates, and the daily averaged values we obtain from the summarized dive profiles show good concordance with those obtained from visually inspected high resolution dive profiles. Compared with the existing approach^41^, which reported 71.4% of retained dives as being true drift dives, our approach retained 87.5%. That gives over a 15% increase in the true drift rate retention, reducing the impact of false positives on the estimated drift rate time series, and contributing to reduce the error/variance of the estimation.

Inclusion of false positive drift dives can result in a higher variance among drift rate estimates and require some further processing; often achieved with smoothing/interpolating techniques such as splines^23,41,59^. Such smoothing/interpolating techniques are based on purely statistical approaches, without any biological process underpinning them. Using a custom Kalman filter incorporates a biologically relevant mechanistic model. Although this filter does not remove every non-drift dive, it greatly reduces their occurrence to approximately 10% of the final set of retained dives. The filter also reduces the variability of the daily drift rate estimates, by over an order of magnitude (Fig. 5) because any accepted non-drift dive (false positive) has to be consistent with the drifting time series of the seal.

An important improvement from previous approaches is that our method can detect when the seal is positively buoyant. Positive buoyancy has implications for quantifying the individual foraging behaviour and success of individuals, as well as the quality of the foraging grounds. In our study, we have processed five post moulting trips, of which three exhibited substantial periods of positive buoyancy up to 150 days, Compiling a realistic record of daily body condition changes would have not been possible with previous approaches which is the ultimate goal of our approach. We make our method available to the research community in the form of an R package under a General Public License.

The results are also consistent with expectations regarding the energy budgets of seals^23,44,59^. All the seals exhibit their lowest body condition at the start of the foraging trip, after fasting for 1–2 months (Appendix C). They show a progressive increase of body condition as they remain at sea, indicating that they are foraging sufficiently well for their physiological needs and to gradually replenish their lipid reserves. In the longer post-moult time-series periods of positive buoyancy occur, often followed by a return to negative buoyancy.

Once buoyancy changes at temporal scales of days to months for individual animals can be estimated these data can be used to relate patterns of individual foraging success to factors such as such as who lives or dies, or who pups successfully, and how this links to where (spatially) and how (functionally) individuals may forage. Compiling patterns of foraging success across individuals will facilitate population level studies such as why some populations are stable and others declining^63–65^. Southern elephant seals have been tagged from all Southern Ocean breeding populations^35^, a global effort spanning more than two decades. Many hundreds of individual animals have been tagged, including both sexes as well as adults and juveniles^66^. Our automated approach is tractable for analysing existing and ongoing large dataset collections for larger overarching studies for example that link performance at-sea to key life-history traits such as survival and reproduction. To date this has been has been difficult given some of the limitations of the analytical tools available to the community.

Marine predators live in a highly heterogeneous seascape, requiring them to make decisions about where to go for their different life activities (e.g. foraging, breeding). Elephant seals are generalist consumers of a wide array of mesopelagic fishes, squid and crustaceans^4^ and the decisions individuals make are likely to be sex-dependent^67^, change ontogenetically^68–71^ and vary regionally^4,35^. Estimating daily changes in body condition are useful for enquiries at a patch-scale, i.e., decisions such as whether to leave a patch in relation to foraging success^22,72^. At a broader scale (regional or basin-scale) we can now directly examine changes in behaviour and performance due to environmental conditions, using covariates recorded either onboard the same tags (i.e. temperature, salinity; e.g.^73^) or synoptic information available from satellite sensors and oceanic models^38,52,74^. As Southern Ocean predators, their foraging success can give an integrated (over time and across space) indication of relative quality of the regions in which they forage^39^. Patterns in body condition can be used to evaluate the spatial distribution of prime foraging areas and their change in response to environmental conditions^38,39,74^. Such a robust metric affords the opportunity to directly pursue ecological questions linking animal ecology, behaviour, physiology and environment.

## Supplementary information

Suplementary material

## Data Availability

Code available at: https://github.com/farcego/slimmingDive.
