# Supplementary material for: A quantitative, hierarchical approach for detecting drift dives and tracking buoyancy changes in southern elephant seals
Source: Sci Rep. 2019 Jun 20;9:8936. doi: 10.1038/s41598-019-44970-1 (PMC6586652; doi:10.1038/s41598-019-44970-1)

Supplementary material for:

**A quantitative, hierarchical approach for detecting drift dives and tracking buoyancy changes in southern elephant seals.**

Fernando Arce ^a,b^, Sophie Bestley ^a,b,d^, Mark A. Hindell^a, d^, Clive R. McMahon ^a,c^ and Simon Wotherspoon^a,b^

^a^ Institute for Marine and Antarctic Studies/ University of Tasmania

^b^ Australian Antarctic Division

^c^ Sydney Institute of Marine Science, Mosman, 2088, New South Wales, Australia

^d^Antarctic Climate and Ecosystems CRC

^*^ Corresponding author: [Fernando.ArceGonzalez@utas.edu.au](mailto:Fernando.ArceGonzalez@utas.edu.au)

**Appendix A: Development and application of threshold criteria**

Candidate drift dives are grouped according to the order in which the inflection points are selected, identified by application of a ‘Reverse’ Broken stick algorithm (8 groups, allocated as positive/negative subgroups, see Methods and Table 3). This Appendix provides information on the development of the set of threshold criteria with respect to dive profile characteristics to apply across groups, to automatically select those dives whose drift rates will be submitted to the subsequent Kalman filtering stage.

**2.1. Description**

The threshold criteria to select drift dives are based on dive proportions generated by studying the general shape of drift dives. Figure 1 in the main text provides a visual aid to the dive segments and variables referred to. The full list of dive variables used to develop threshold criteria are:

**BSA depth ratios (d1, d2, d3, d4):** ratio between the depths of the first (d1), second (d2), third (d3) and fourth (d4) inflection points and the maximum depth, i.e. d1 = D1/MaxDepth etc.

This ratio generates a measure of dispersion of the BSM points with respect to the maximum depth. Their relationship depends on the shape of the dive. In negative drift dives, the ratio of the start point of the drift segment should not be one (or close to) as the seal must keep descending due to its negative buoyancy (maximum depth should be reached at the end of the drifting segment). For positive drift dives, the ratio of the end point of the drift segment should again not be close to one. The seal is expected to have reached the maximum depth before starting to drift upwards, and as the seal is ascending the water column during the drift phase; the depth at the end of the drift segment should be shallower than the maximum depth.

**meand:** mean value of the four BSM depth ratios (d1, d2, d3, d4)

The mean value of the depth ratios should not be close to one. As the drift segment should start (positive drift dive) or end (negative) at or close to the maximum depth, and will cover the smoothest part of the dive trajectory (i.e., least variation in depth over time), the rest of the inflection points contain the complexity of the dive activity, which should not occur close to the maximum depth.

**sratio:** ratio between the vertical rate of the descending segment and the vertical rate of the first segment post-descent, i.e. [∆(D_1_)/∆(T_1_)] / [∆(D_2_)/∆(T_2_)].

For negatively buoyant seals drifting during the first segment, values lower than 1 are rejected; because, it is not expected for an animal to exhibit an active descent with a rate of depth change slower than occurs during the drift segment. This ratio should also depart from 1, since this would point to a ‘V’ shaped dive. On the other hand, values too high are characteristic of a flat post-descent segment, typical of ‘U’, or square-bottomed dives.

**sdd:** standard deviation of the four BSM depth ratios (d1, d2, d3, d4).

The standard deviation of the depth ratios should not be too small (indicating that all the inflection points have occurred at a similar depth) or too large (indicating a complex dive profile).

**BSM point residuals (r1, r2, r3, r4):** residuals obtained from fitting a linear regression model through the four BSM points. The residual of the start or end points of a drift segment should be expected to be negative for certain groups (indicated in Table 3).

**mdepthbias:** Difference between the time at maximum depth (T1) and half of the total dive duration. This should be positive for negative drift dives as the maximum dive depth occurs at the end of the drift segment, in the second half of the dive; and conversely negative for positive drift dives as the maximum dive depth should occur at the start of the drift segment, in the first half of the dive.

**mdepthr:** Ratio between the averaged depth of the BSM points and the maximum depth. Values close to 1 would be indicating small changes in depth along the dive, not to be expected in drift dives. Large values would indicate ‘V’ shaped dives.

**BSM segment lengths (hp1, hp2, hp3):** Euclidean length of the three dive segments, determined by the 4 BSM points. For two segments with the same duration, the segment with a larger change in depth will have a larger value.

**Proportional duration of the BSM diving segments (ps1, ps2, ps3):** proportion of the dive duration spent in the three segments defined by the BSM points. For a given drift segment, it should be reasonably large

**Avratio:** Deviation of the time at which maximum depth occurs, with respect to half of the dive duration (i.e., the midpoint of the dive). It is useful to determine the drift segment in some cases.

**Occurrence of the inflection point with respect to the dive duration (t1, t2, t3, t4):** i.e., t1 = T1 / Dive duration, t2 = T2 / Dive duration and so on. Here T1 refers to the time (in seconds) since the start of the dive at which the first inflection point occurred, and so on.

**Mrratio:** ratio between the length of the fourth (last) residual of the BSM and the maximum dive depth.

**2.2 Application**

All criteria are simultaneously compared between drift and non-drift dives for each of the 15 subgroups (8 negative and 7 positive). We constructed density plots of the dive variables with the overlapping area between drift and non-drift dives shaded in dark (Figure A2.1). We made plots for each seal and for the three seals altogether to maximize removal of non-drift dives while minimizing removal of drift dives, thereby balancing the optimum threshold among the three seals.

Using these plots, the criterion showing less overlap between the density distribution of drift and non-drift dives was chosen and investigated in detail to choose the numerical threshold to maximize the rejection of non-drift dives while minimizing the rejection of true drift dives (Figure A2.2). For that purpose, the values considered reasonable were a reduction of around 50% (at least) of non-drift dives at the cost of up to around a 5% of the drift dives.

This procedure follows sequentially through the relevant criteria, we show two more examples below: for criteria ps1 (Figure A2.3 and 2.4) and t1 (Figure A2.5 and 2.6). The final number and percentages of retained certain drift dives across the eight major groups of candidate drift dives are given in Appendix B.

**Figure A2.1. First example showing the application of the dive criteria and choice of threshold.** Here, for negative drift dives of the group defined by the ifp 2.1.4.3. Once all density plots are visualized, the criteria showing less overlap between drift/non-drift dives is chosen: d1 in this example.


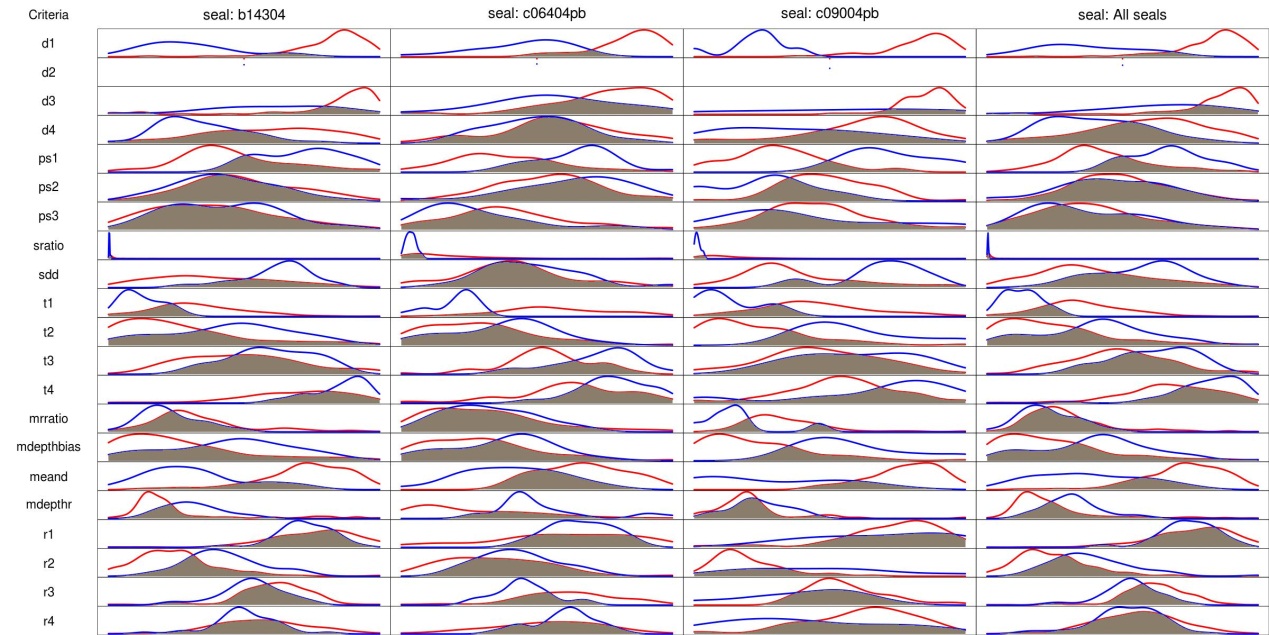


**Figure A2.2. A reject-accept plot generated following the choice of criteria (here d1, from Figure A2.1).** This shows the proportion of accepted drift and non-drift dives along a gradient in the threshold values is generated with the three seals, both independently and pooled.


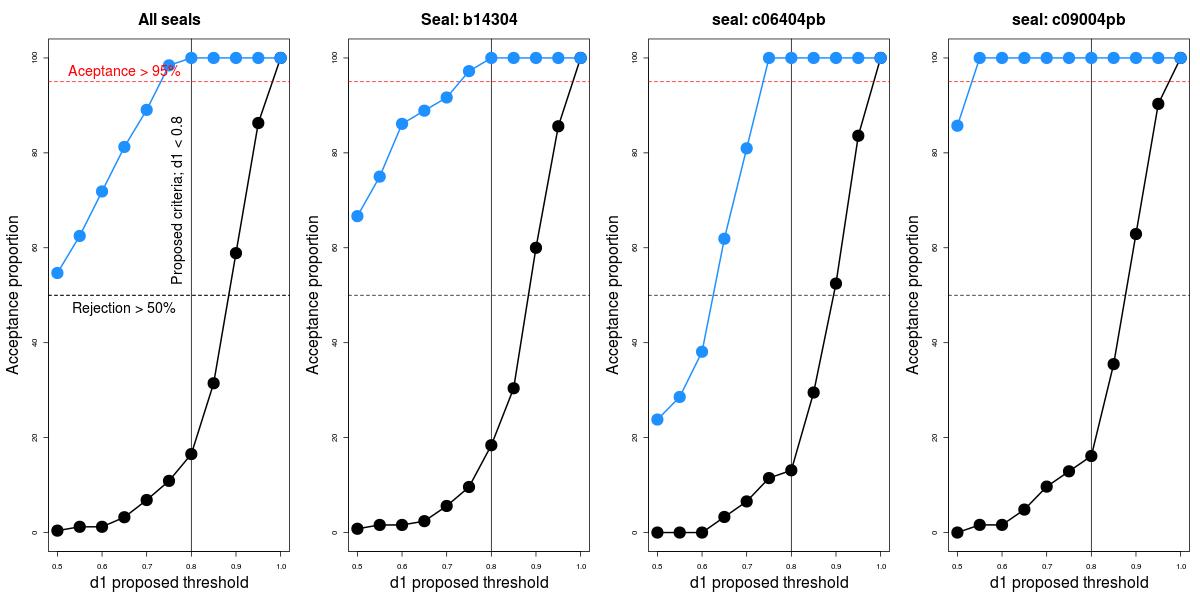


**Figure A2.3. Second example showing the application of the dive criteria and choice of threshold.** Following the choice of d1 above, ps1 in this example. Note that while previous criteria (d1) could have been optimized more for seals b14304 and c09004pb, it would have had a large negative impact on seal c064404pb.


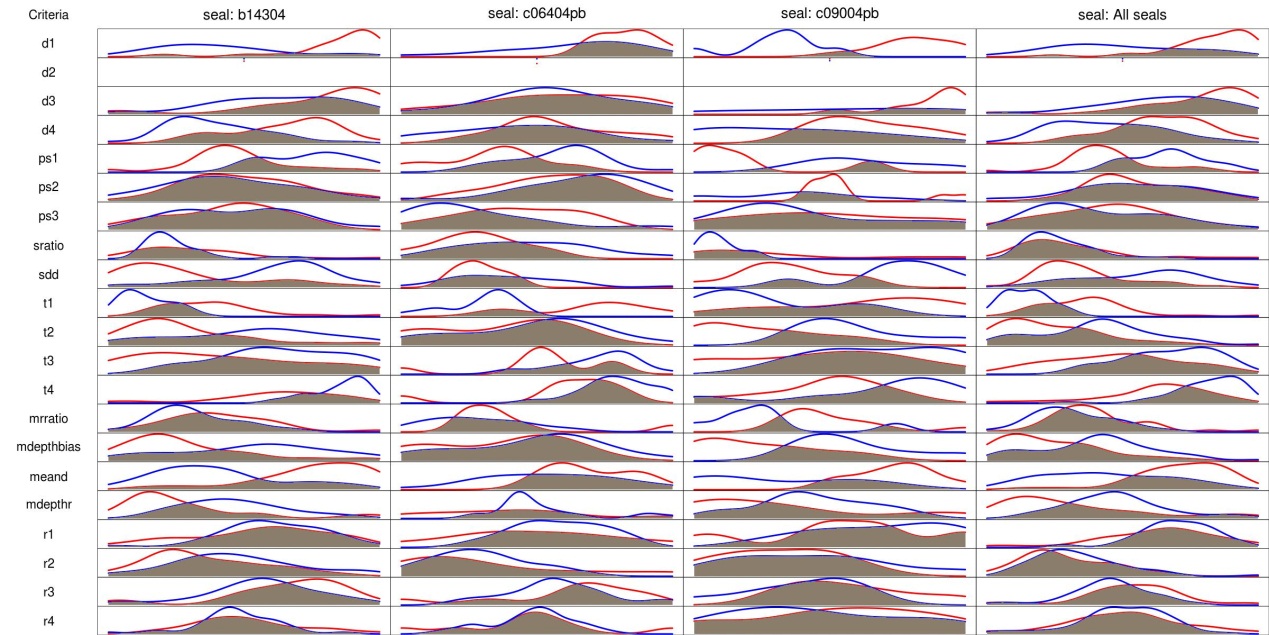


**Figure A2.4. A reject-accept plot generated following the choice of criteria (here ps1, from Figure A2.3).**


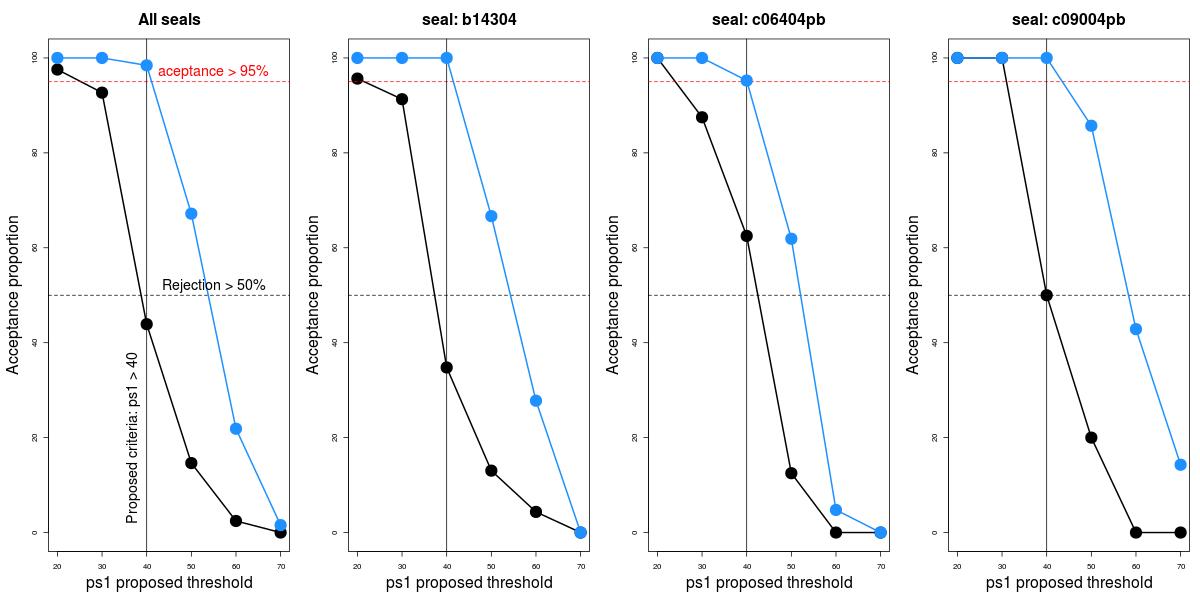


**Figure A2.5. Third example showing the application of the dive criteria and choice of threshold.** Following the choice of d1 and ps1 above, t1 in this case.


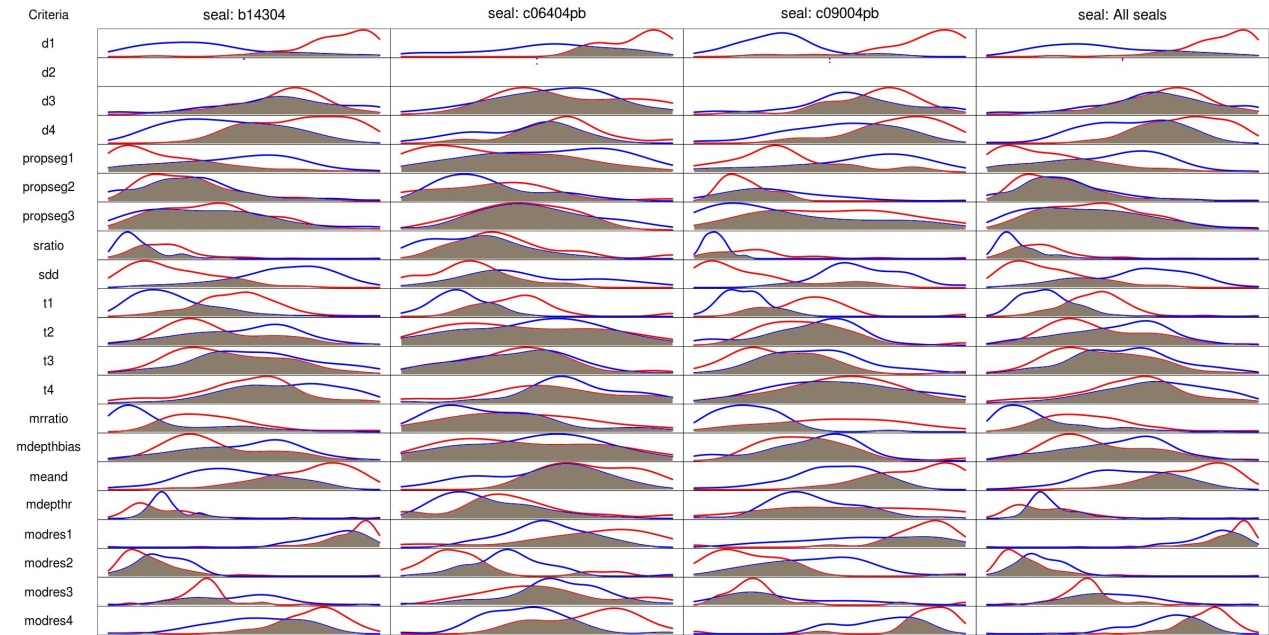


**Figure A2.6. A reject-accept plot generated following the choice of criteria (here t1, from Figure A2.5).**


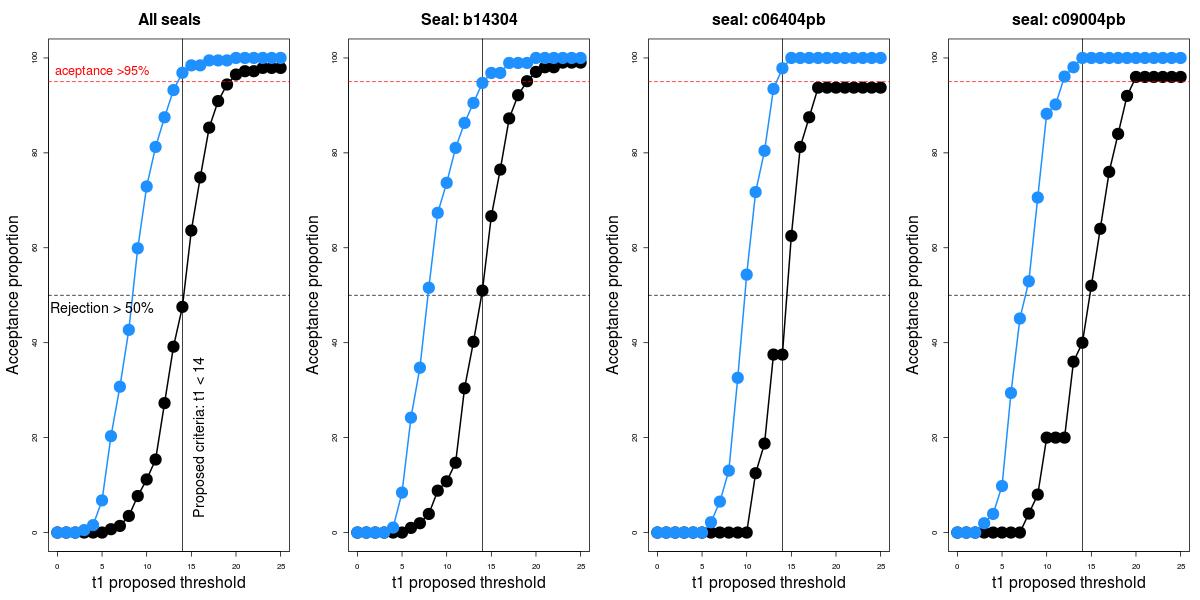


**Appendix B.** Visual dive classification results for each visually inspected seal associated with each inflection point order generated with the reverse broken stick algorithm (RBSA). Dive types: 1 – not a drift dive, 2 – certain negative drift dive, 3 – uncertain negative drift dive, 4 – certain positive drift dive, 5 – uncertain positive drift dive. The eight major groups retained for generating the set of candidate drift dives appear in bold, with the number and percentages for the retained certain drift dives occurring within these groups also given below.

| Seal Id → | b14304pm | | | | | c06404pb | | | | | c09004pb | | | | |
| --- | --- | --- | --- | --- | --- | --- | --- | --- | --- | --- | --- | --- | --- | --- | --- |
| Dive Type → | 1 | 2 | 3 | 4 | 5 | 1 | 2 | 3 | 4 | 5 | 1 | 2 | 3 | 4 | 5 |
| RBSM group ↓ |  |  |  |  |  |  |  |  |  |  |  |  |  |  |  |
| 1.2.3.4 | 8 |  |  |  |  | 2 |  |  |  |  | 2 |  |  |  |  |
| 1.2.4.3 | 10 |  |  |  |  | 2 | 2 |  |  |  | 1 |  |  |  |  |
| 1.3.2.4 | 49 | 1 |  | 2 |  | 8 |  |  |  |  | 8 |  |  |  |  |
| 1.3.4.2 | 418 |  |  | 2 | 1 | 75 |  |  |  |  | 41 |  |  |  |  |
| 1.4.2.3 | 83 |  |  |  | 1 | 30 |  |  |  |  | 24 |  |  |  |  |
| 1.4.3.2 | 149 |  |  |  | 1 | 24 |  |  |  |  | 10 |  |  |  |  |
| **2.1.3.4** | **270** | **36** | **7** | **6** | **17** | **98** | **7** |  |  |  | **94** | **21** | **4** |  |  |
| **2.1.4.3** | **864** | **110** | **8** | **4** | **6** | **183** | **53** | **1** |  |  | **175** | **48** | **2** |  |  |
| 2.3.1.4 | 196 | 3 | 1 | 1 | 1 | 63 | 2 |  |  |  | 20 |  |  |  |  |
| 2.3.4.1 | 163 | 3 | 4 |  |  | 22 |  |  |  |  | 14 | 2 |  |  |  |
| **2.4.1.3** | **1775** | **27** | **4** | **7** | **7** | **653** | **8** | **2** |  |  | **488** | **6** | **2** |  |  |
| 2.4.3.1 | 509 | 4 | 5 |  |  | 70 | 1 |  |  |  | 66 |  | 1 |  |  |
| **3.1.2.4** | **562** | **9** | **1** | **85** | **9** | **372** | **16** | **2** |  |  | **378** | **22** | **4** |  | **6** |
| **3.1.4.2** | **2589** | **73** | **14** | **34** | **15** | **916** | **16** |  |  |  | **619** | **14** | **6** |  |  |
| **3.2.1.4** | **109** | **86** | **5** |  |  | **27** | **23** |  |  |  | **19** | **9** | **1** |  |  |
| 3.2.4.1 | 214 | 9 | 5 |  |  | 62 | 11 |  |  |  | 29 | 2 | 1 |  |  |
| **3.4.1.2** | **608** | **4** | **1** | **11** | **4** | **311** | **6** |  |  |  | **149** | **2** | **2** |  |  |
| 3.4.2.1 | 29 | 3 | 1 |  |  | 2 | 2 |  |  |  | 4 |  |  |  |  |
| 4.1.2.3 | 77 | 2 | 1 | 9 |  | 30 |  |  |  |  | 32 |  |  |  |  |
| 4.1.3.2 | 258 | 21 | 3 | 2 | 3 | 62 | 1 |  |  |  | 35 |  |  |  |  |
| **4.2.1.3** | **676** | **141** | **26** | **7** | **5** | **399** | **36** | **1** |  | **1** | **261** | **64** | **9** |  |  |
| 4.2.3.1 | 105 | 2 | 2 | 1 | 1 | 22 | 1 |  |  |  | 15 | 1 |  |  |  |
| 4.3.1.2 | 285 | 8 |  | 8 | 3 | 245 | 6 |  |  |  | 105 | 8 | 3 |  |  |
| 4.3.2.1 | 24 |  |  |  |  | 3 |  |  |  |  |  | 1 |  |  |  |
| Total | 10030 | 542 | 88 | 179 | 74 | 3681 | 191 | 6 | 0 | 1 | 2589 | 200 | 35 | 0 | 6 |
| Retained |  | 486 |  | 154 |  |  | 181 |  |  |  |  | 186 |  |  |  |
| Percentage (%) |  | 90 |  | 86 |  |  | 95 |  |  |  |  | 93 |  |  |  |

**Appendix C. Visual representation of the complete filtering process.**

Each seal (n = 13) is in a row, with seals used for developing the method occupying the first three rows. First column: the subset of candidate drift dives based on the RBSA. At this stage, the drift segment is identified and drift rate calculated. Second column: dives remaining after the threshold criteria are applied across groups. Third column: final output after the Kalman filter where x-axis represents days at sea and y-axis represents drift rate. The umbers and proportions of dives retained at each step for the validation seals are available at Table 4 on the manuscript.


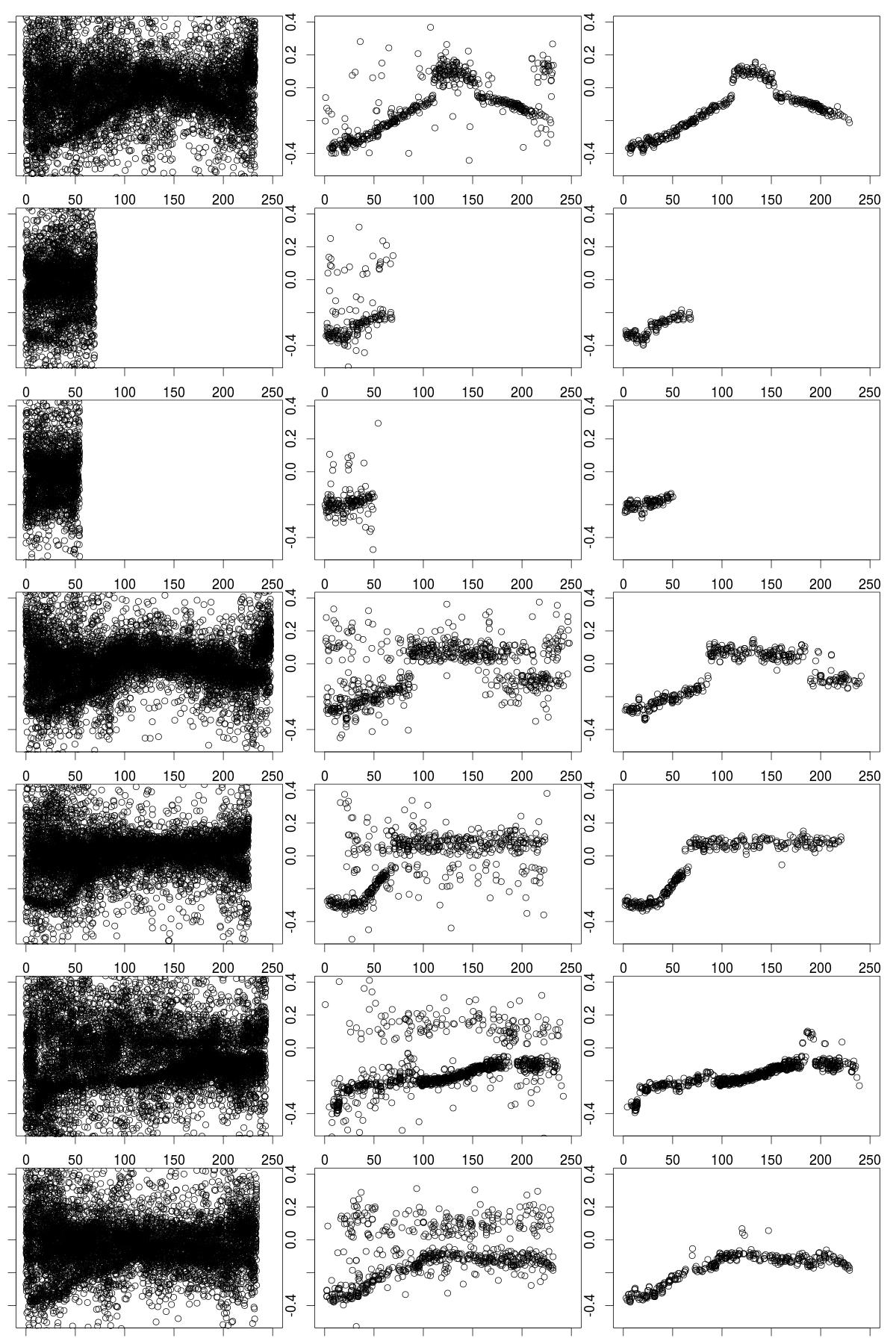


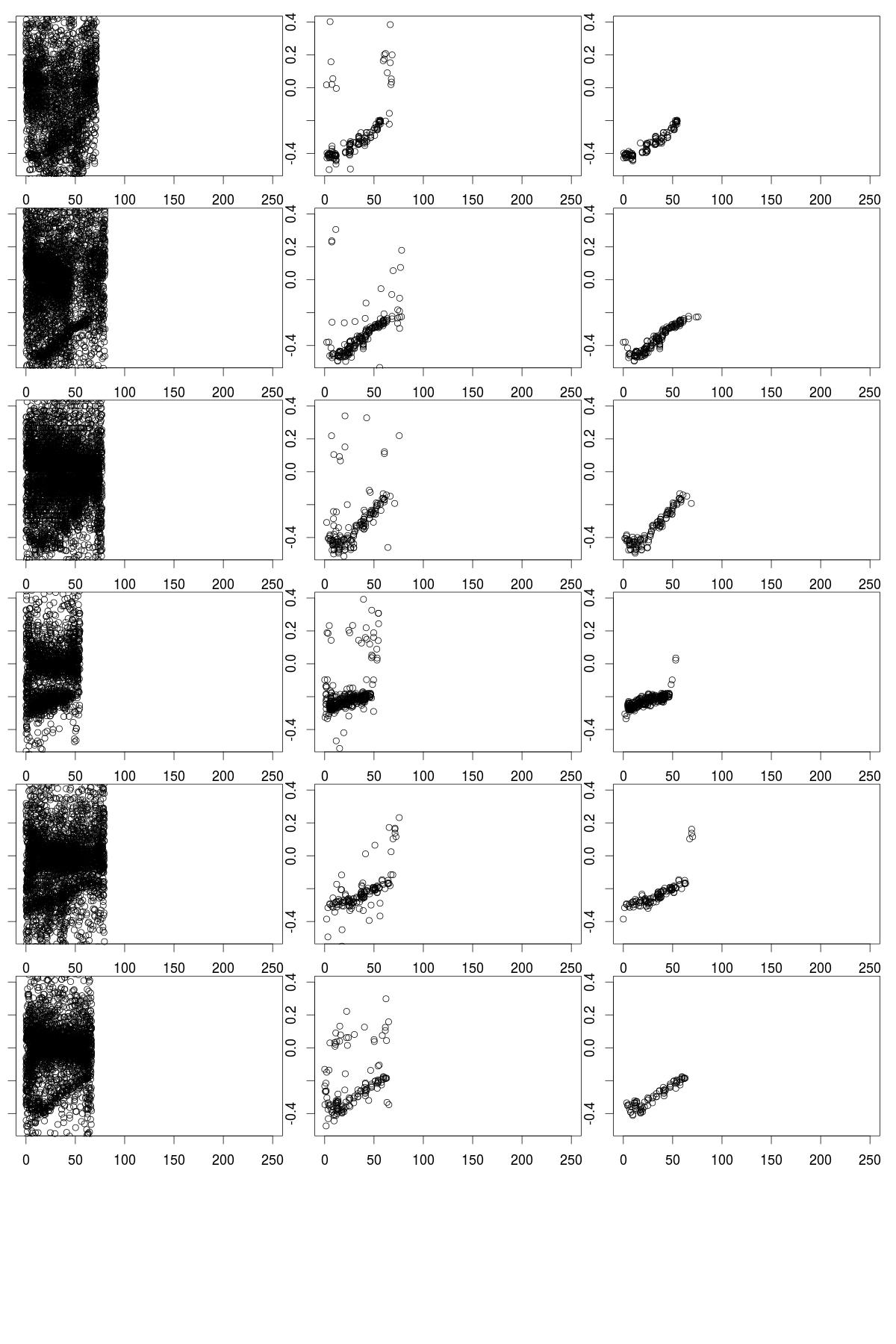

Supplement: Supplementary file 1 — Suplementary material [file 41598_2019_44970_MOESM1_ESM.docx]
